# The Use of Retinal Microvascular Function and Telomere Length in Age and Blood Pressure Prediction in Individuals with Low Cardiovascular Risk

**DOI:** 10.3390/cells11193037

**Published:** 2022-09-28

**Authors:** Hala Shokr, Victoria Lush, Irundika HK Dias, Anikó Ekárt, Gustavo De Moraes, Doina Gherghel

**Affiliations:** 1 Vascular Research Laboratory, College of Health and Life Sciences, Aston University, Birmingham B4 7ET, UK; 2 Pharmacy Division, Faculty of Biology, Medicine and Health, University of Manchester, Manchester M13 9PL, UK; 3 Computer Science, School of Informatics and Digital Engineering, College of Engineering and Physical Sciences, Aston University, Birmingham B4 7ET, UK; 4 Aston Medical School, College of Health and Life Sciences, Aston University, Birmingham B4 7ET, UK; 5 Bernard and Shirlee Brown Glaucoma Research Laboratory, Department of Ophthalmology, Columbia University Irving Medical Center, New York, NY 10032, USA; 6 Division of Cardiovascular Sciences, University of Manchester, Manchester M13 9PL, UK

**Keywords:** biological age, telomeres, prediction, blood pressure, regression

## Abstract

Ageing represents a major risk factor for many pathologies that limit human lifespan, including cardiovascular diseases. Biological ageing is a good biomarker to assess early individual risk for CVD. However, finding good measurements of biological ageing is an ongoing quest. This study aims to assess the use retinal microvascular function, separate or in combination with telomere length, as a predictor for age and systemic blood pressure in individuals with low cardiovascular risk. In all, 123 healthy participants with low cardiovascular risk were recruited and divided into three groups: group 1 (less than 30 years old), group 2 (31–50 years old) and group 3 (over 50 years old). Relative telomere length (RTL), parameters of retinal microvascular function, CVD circulatory markers and blood pressure (BP) were measured in all individuals. Symbolic regression- analysis was used to infer chronological age and systemic BP measurements using either RTL or a combination of RTL and parameters for retinal microvascular function. RTL decreased significantly with age (*p* = 0.010). There were also age-related differences between the study groups in retinal arterial time to maximum dilation (*p* = 0.005), maximum constriction (*p* = 0.007) and maximum constriction percentage (*p* = 0.010). In the youngest participants, the error between predicted versus actual values for the chronological age were smallest in the case of using both retinal vascular functions only (*p* = 0.039) or the combination of this parameter with RTL (*p* = 0.0045). Systolic BP was better predicted by RTL also only in younger individuals (*p* = 0.043). The assessment of retinal arterial vascular function is a better predictor than RTL for non-modifiable variables such as age, and only in younger individuals. In the same age group, RTL is better than microvascular function when inferring modifiable risk factors for CVDs. In older individuals, the accumulation of physiological and structural biological changes makes such predictions unreliable.

## 1. Introduction

Ageing is no longer perceived as a “disease” but as a physiological phenomenon. Still, ageing does represent a major risk factor for many pathologies that limit human lifespan, including cardiovascular diseases (CVD) [1,2,3]. It has been, however, admitted that individuals do not age at a similar pace [4]. Indeed, according to the so-called “remodelling theory of ageing”, differences in genetic and environmental factors result in a large variety of individual operational decline of biological systems as well as in the capacity of cells and systems to adapt to such changes [5,6]. This observation has led to the concept of “biological ageing”, which represents a measure of each individual’s bodily functional downturn [4], as opposed to chronological ageing, which is just the passage of time.

Biological age correlates, in part, with its chronological counterpart; however, as opposed to the later, its precise measurement could be a good candidate to identify individuals that are at higher risk of various pathologies, traditionally related to ageing [7]. This is of interest because multifactorial disorders such as CVD have a high individual variability in terms of susceptibility, onset and progression and, therefore, risk calculators validated only in a few population groups suffer from many limitations [8,9,10].

Finding good measurements of biological ageing that are also good risk markers for CVD is still an ongoing process, though many markers are showing a strong potential [11,12]. Among those, reduced leukocyte telomere length, beside its “age-predictive” power, has also been associated with known CVD risk factors, such as positive family history, abnormal blood pressure (BP) [13], lower high-density lipoprotein cholesterol (HDL-C) levels [14], smoking, alcohol consumption [15,16], high levels of oxidative stress [17] and increased endothelial cell turnover [18]. In addition, microvascular function is also affected by ageing and the presence of CVD risk [4,19,20,21,22,23,24]. All these qualities could catapult these two parameters to the forefront of the list of candidates for individual CVD risk prediction and, possibly, for the assessment of biological ageing. Nevertheless, the question still remains whether the quality of such predictions can be increased by using a combination of these two biomarkers.

In order to determine that, simple predictions such as those for chronological ageing and early markers for CVD risk such as systemic BP, would need to be first tried. Therefore, the aim of the present study was to use RTL and microvascular function (assessed at the retinal level), either separately or in combination, to infer chronological age and systemic BP in healthy individuals with low CVD risk.

## 2. Materials and Methods

### 2.1. Study Participants

Healthy individuals aged 18 years old and above were recruited for this study through advertisements at the Vascular Research Laboratory, Aston University (Birmingham, UK). Ethical approval was sought from the relevant local ethics committees, and written informed consent was received from all participants prior to study enrolment. The study was designed and conducted in accordance with the tenets of the Declaration of Helsinki, and all study-related procedures adhered to institutional guidelines.

Study exclusion criteria were defined as the positive diagnosis of CVD, cerebrovascular disease, peripheral vascular disease, severe dyslipidemia (defined as plasma triglycerides > 6.00 mmoL/L or cholesterol levels > 7.00 mmoL/L), diabetes as well as other metabolic disorders or chronic diseases that required treatment. Individuals treated for systemic hypertension as well as those using any vasoactive medications such as dietary supplements containing vitamins or antioxidants and bronchodilators were also excluded from the study. Potential participants were also screened for ocular diseases and were excluded from the study if they had a refractive error of more than ±3DS and more than ±1DC equivalent, intra-ocular pressure (IOP) greater than 21 mmHg, cataract, or any other media opacities, as well as history of intra-ocular surgery or any form of retinal or neuro-ophthalmic disease affecting the ocular vascular system. Individuals with sings of hypertensive retinopathy at the initial fundus examination were also excluded.

### 2.2. General Investigations

Standard anthropometric measures of height and weight were recorded to determine body mass index (BMI = weight/height). Systolic blood pressure (SBP), diastolic blood pressure (DBP) and heart rate (HR) were measured using an automatic Blood Pressure monitor (UA-767; A&D Instruments Ltd., Abingdon, UK) to determine mean arterial pressure (MAP = 2/3 DBP + 1/3 SBP). IOP readings were obtained using non-contact tonometry (Pulsair; Keeler Ltd., Windsor, UK).

### 2.3. Blood Analyses

Blood and plasma samples drawn from the antecubital fossa vein were assessed immediately for fasting glucose (GLUC), triglycerides (TG), total cholesterol (T-CHOL) and high-density lipoprotein cholesterol (HDL-C) using the Reflotron Desktop Analyzer (Roche Diagnostics, Welwyn Garden City, UK). Low-density lipoprotein cholesterol (LDL-C) values were calculated as per the Friedewald equation [25]. These variables, in addition to the above parameters, were used to calculate the Framingham Risk Score (FRS) for each individual [26].

Absolute CVD risk percentage over 10 years was classified as low risk (<10%), intermediate risk (10–20%) and high risk (>20%) [27].

In addition, glutathione recycling assays (oxidized (GSH) and reduced (GSSG)) were also performed, as detailed previously [28]. Briefly, a 30 μL aliquot of EDTA blood was pretreated with 33.3 μL of 100 mg/mL 5-sulfosalicylic acid (SSA), 936.7 μL sodium phosphate buffer (pH 7.5) to release GSH via cellular disruption and protein precipitation. The sample was centrifuged at 13,000 rpm for 5 min, and the supernatant was stored at −80 °C for further analyses. Based on previous reports of sample stability, assays were conducted within 2 months of collection [29]. The GSH levels [t-GSH − (2 × GSSG)] and the redox index (defined as the GSH/GSSG ratio) were determined according to an established enzymatic recycling assay [30,31].

### 2.4. Framingham Risk Score (FRS) Calculation

The FRS for each individual was calculated according to already published formula [32]. All screened individuals were classified, using the absolute CVD risk percentage over 10 years, as low risk (<10%), intermediate risk (10–20%) and high risk (>20%) [33]. Only individuals with an FRS < 10% were included in the final analysis.

### 2.5. Dynamic Retinal Microvascular Function Vessel Analysis

**Figure 1:**
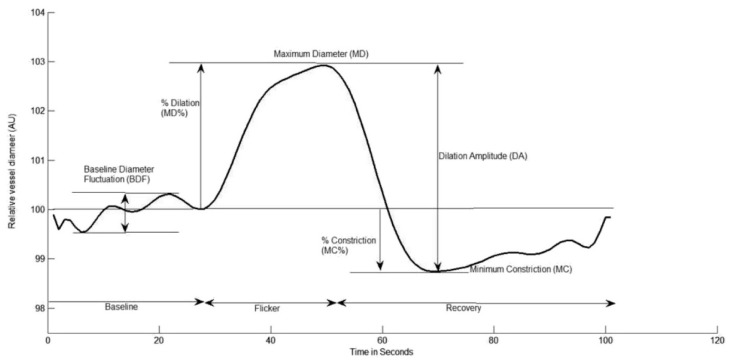
Graphical presentation of the dynamic vessel response profile displaying the parameters calculated and used in analysis. (DA) calculated as (MD-MC). (MD%) calculated as the percent increase from baseline to MD. (MC%) calculated as the percent constriction below baseline following MD.

Retinal microvascular function was assessed using the dynamic retinal vessel analyser (DVA, IMEDOS GmbH, Jena, Germany) in accordance with an established protocol [34] Using a validated in-house algorithm, the following vessel reactivity and time-course parameters were determined: the average baseline diameter and range of maximum and minimum baseline vessel diameters (baseline diameter fluctuation, BDF); the maximum vessel dilation diameter during flicker stimulation expressed as a percentage change relative to baseline diameter (MD%) and the time taken in seconds to reach the maximum diameter (tMD); the maximum vessel constriction diameter during the post-flicker recovery period expressed as a percentage change relative to baseline diameter (MC%) and the time taken in seconds to reach the maximum vessel constriction diameter (tMC); the overall dilation amplitude (DA) calculated as the difference between MD and MC; and the baseline-corrected flicker response (BCFR) used to describe the overall dilation amplitude after normalizing for fluctuations in baseline diameters (DA-BDF). In addition, the arterial (A) and venous (V) dilation slopes (SlopeAD/VD = (MD − baseline diameter)/tMD) and constriction slopes (SlopeAC/VC = (MC − MD)/tMC) were also calculated (Figure 1) [35,36].

The reproducibility and sensitivity of the RVA in healthy subjects have been described previously [37,38].

### 2.6. Relative Telomere Length (RTL) Assessment

DNA extractions were performed using the QIAamp DNA Mini Kit (Qiagen, Machester, UK) according to the manufacturer’s protocol [39]. DNA purity was detected by NanoDrop™ 1000/c (Spectrophotometers, Thermo Fisher Scientific, Waltham, MA USA) and samples were stored at −80 °C until further analysis. Relative telomere length (RTL) was measured using real-time polymerase chain reaction (RT-PCR) according to a previously published method [40] using LightCycler^®^ 480 Instrument (Roche Diagnostics GmbH, Mannheim, Germany). Briefly, primers for telomere repeats and a normalizing genomic sequence (Table 1) was prepared in a 25 μL PCR reaction, consisting of Precision 2× qPCR Mastermix (0.025 U/μL Taq polymerase, 5 mM MgCl2, dNTP mix 200 μM each dNTP) and 15 ng of template DNA. Samples for both the telomere and single-copy gene amplifications were performed in triplicate with non-template control. The ratio of telomere to the normalizing genomic control sequence (T/S ratio) was calculated as previously described [40,41] to provide an indication of RTL.

### 2.7. Sample Size and Analysis

Based on previous studies, a change of 30% with an SD of 2.5% in retinal vessels reactivity was shown to be significant [42,43]. As the study design was multifactorial in nature, it was calculated that a total sample size of *n* = 120 was sufficient to provide 95% power at an alpha level of 0.05.

#### 2.7.1. Statistical Analysis

All analyses were performed using Statistica^®^ software (version 13.3, StatSoft Inc., Tulsa, OK, USA). Distributions of continuous variables were determined by the Shapiro–Wilk test. In cases where normality of the data could not be confirmed, appropriate data transformations were made or non-parametric statistical alternatives were used. Univariate associations were determined using Pearson’s (normally distributed data) or Spearman’s method (non-normally distributed data), and forward stepwise regression analyses were performed to test the influence of clinical parameters and circulating markers on the measured vascular reactivity variables. Differences between groups were subsequently assessed using one-way ANOVA or ANCOVA, as appropriate, followed by Tukey’s post hoc analysis; *p*-values of <0.05 were considered significant, except in certain cases where a stricter *p*-value of <0.01 was adopted to correct for multiple comparisons [44,45,46].

#### 2.7.2. Symbolic Regression-Based Analysis

As all our study’s measurements represent numerical values and the relationships between these values are unknown and, possibly, of a nonlinear nature, symbolic regression [47,48,49] was also used to assess inter-variable relationships. While classical regression methods rely on a priori definition of the model structure and only provide parameters for the pre-selected models, symbolic regression derives both the model structure and its parameters automatically. Furthermore, symbolic regression is rapidly gaining popularity in various research fields due to the interpretability of models that it generates [50,51].

Models were generated for each age group separately, therefore ensuring an equitable consideration of all ages without the need to apply data augmentation methods to generate synthetic data. For each age group, we compared predicted age and systemic BP based on the 3 generated models: 1. combined RTL and artery measurements; 2. artery measurements only and; 3. RTL only. K-fold cross-validation and mean absolute error (MAE) were used for assessing model quality.

## 3. Results

One hundred and twenty-three healthy participants with low global cardiovascular risk (<10% at 10 years as assessed by the FRS) and similar dietary habits were included in the final analyses.

We divided our study participants into three age groups (30 years and below, between 31 and 50 years and over 50 years of age). There were no statistically significant differences between the number of participants in each group (*p* > 0.05). In addition, the number of men and women in each group was similar (*p* > 0.05).

Table 1 shows the general and circulatory markers characteristics of the study population stratified by age groups. 

The three groups were also similar with regards to HR, BMI, TG, HDL-C, GSH and GSSG (all *p* value > 0.05). There were statistically significant differences in SBP (*p* = 0.0129) and DBP (*p* = 0.0022), with older individuals displaying, as expected, higher values. In addition, although still within the normal range, T-CHOL and LDL-C plasma concentrations were significantly different between the three study groups, with middle age and elderly groups showing higher concentrations compared to the younger group (*p* < 0.001 and *p* = 0.001, respectively). Moreover, and as expected, RTL decreased significantly with age (*p* = 0.010).

### 3.1. Differences in Retinal Vascular Function

Group differences in flicker-induced retinal arterial diameter changes (DVA) are summarized in Table 2. All reported values are based on data averaged across the three flicker cycles, with the artery and vein regarded separately.

After controlling for influential covariates identified in multivariate analysis, there were no significant group differences in baseline diameter, BDF, DA, BCFR, MD, MD%, MC, SlopeAD and SlopeAc. There were, however, significant group differences in arterial tMD (*p* = 0.005), MC (*p* = 0.007) and MC% (*p* = 0.010) (Table 2). Post hoc comparisons showed tMD and tMC to be significantly higher in the oldest group when compared to youngest and middle age groups (*p* = 0.012, 0.013 and *p* = 0.0024, 0.0004 respectively). Additionally, artery MC% was also increased in the oldest age group compared to the youngest and middle age groups (*p* = 0.0085 and *p* = 0.0148, respectively). There were no statistically significant differences between the measured venous retinal parameters in all of the study groups.

### 3.2. Correlation Results

RTL was negatively correlated with SBP only in groups 2 and 3 (*p* = 0.033, r = −0.315 and *p* = 0.0116, r = −0.238, respectively). Moreover, there was statistically significant positive correlation between RTL and artery baseline diameter (*p* = 0.039, r = 0.346), BDF (*p* = 0.043, r = 0.318), MC (*p* = 0.032, r = 0.323) and DA (*p* = 0.044, r = 0.338) only in group 3.

### 3.3. Symbolic Regression-Based Analysis

**Figure 2:**
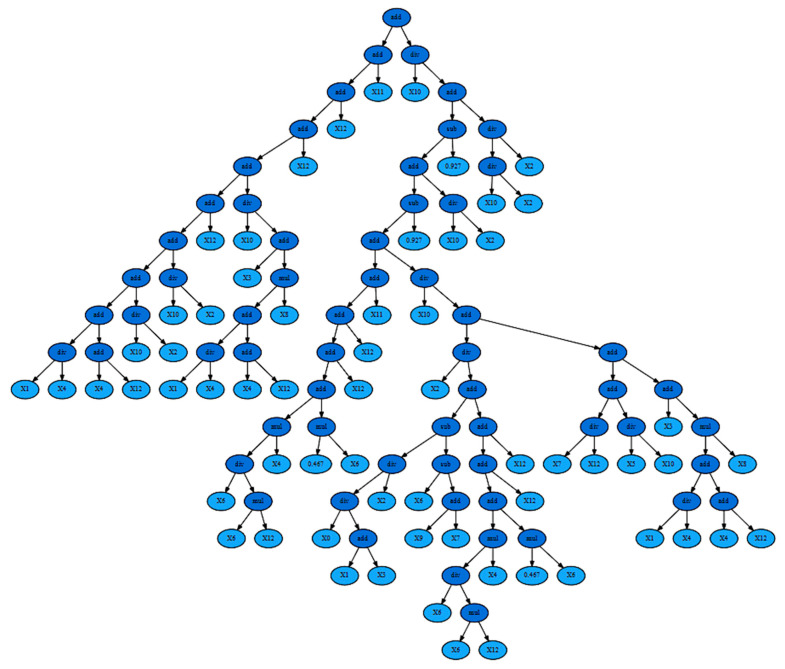
Symbolic Regression tree for age group 1, telomere + arteries experiment (MAE 1.157639). Corresponding expression formula: add(add(add(add(add(add(add(add(add(div(X1, X4), add(X4, X12)), div(X10, X2)), div(X10, X2)), X12), div(X10, add(X3, mul(add(div(X1, X4), add(X4, X12)), X8)))), X12), X12), X11), div(X10, add(sub(add(sub(add(add(add(add(add(mul(div(X6, mul(X6, X12)), X4), mul(0.467, X6)), X12), X12), X11), div(X10, add(div(X2, add(sub(div(div(X0, add(X1, X3)), X2), sub(X6, add(X9, X7))), add(add(add(mul(div(X6, mul(X6, X12)), X4), mul(0.467, X6)), X12), X12))), add(add(div(X7, X12), div(X5, X10)), add(X3, mul(add(div(X1, X4), add(X4, X12)), X8)))))), 0.927), div(X10, X2)), 0.927), div(div(X10, X2), X2)))).

As the differences between groups in retinal microvascular function were mainly perceived at the retinal arteriolar level, only arterial measurements have been used in our symbolic regression analysis. An example age prediction tree generated by the symbolic regression model, and its corresponding expression formulas, is shown in Figure 2. Table 3 presents the symbolic regression’s encodings for each of the retinal arterial parameters (assessed using the DVA instrument), as well as for the RTL.

**Figure 3:**
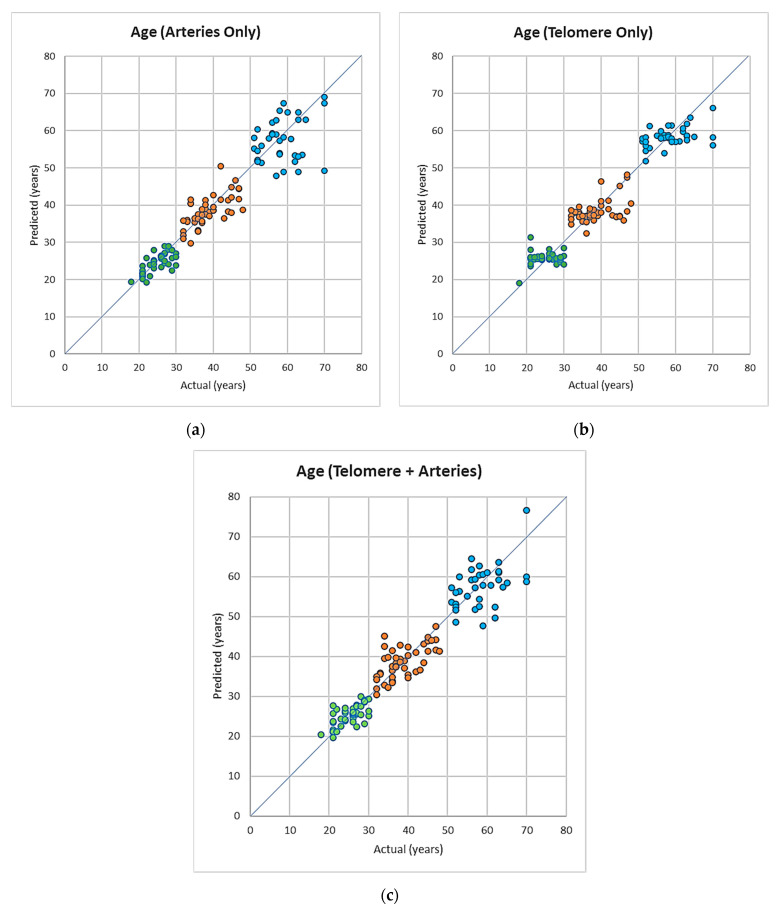
(**a**–**c**) Predicted age vs. actual age using models based on artery data, telomere relative expression and artery and telomere combined data, colour coded for the different age groups (green: 18–30, orange: 31–50, blue: 50+).

The mean absolute error (MAE) values for each age group are presented in Table 4, Table 5 and Table 6 and Figure 3. 

**Age prediction**

In group 1, the error between predicted versus actual values for age are smallest in the case of using artery measurements only, followed by artery and telomeres, while the largest error occurred when the using telomere only. This difference was overall statistically significant (*p* = 0.039, and *p* = 0.045, Table 4a). In group 2, there were no statistically significant differences between MAE using either of predictors *p* > 0.05, Table 5b). In group 3, where using telomere measurements led to the lowest MAE value, however, this was not statistically significant (*p* > 0.05, Table 6c).

**Blood pressure prediction:**

**Figure 4:**
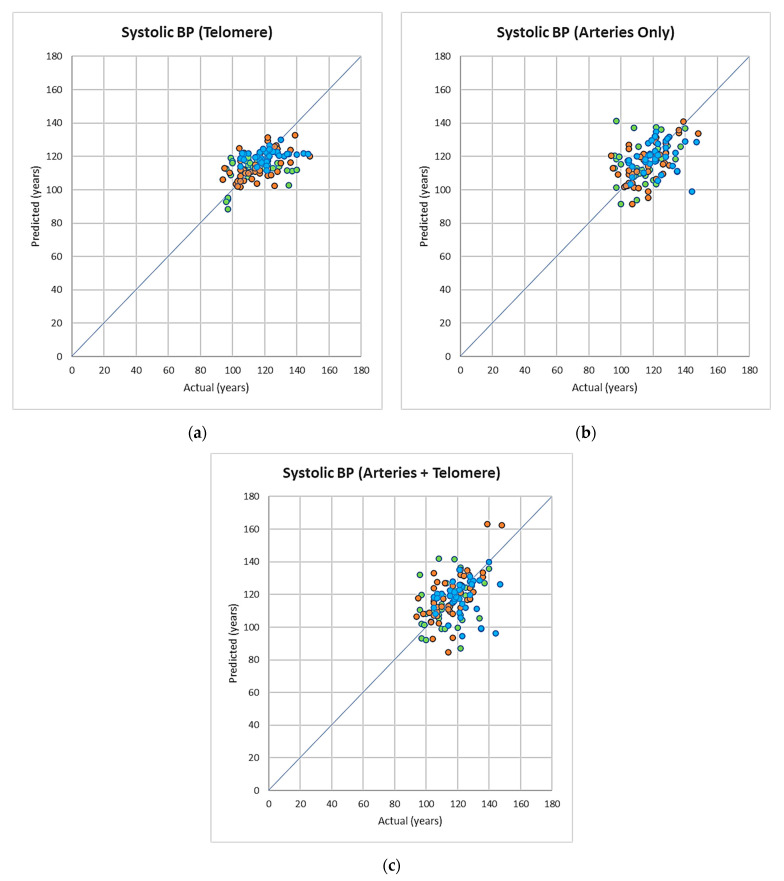
(**a**–**c**) Predicted systolic blood pressure vs. actual systolic blood pressure using models based on artery, telomere, artery + telomeres combined data, colour coded for the different age groups (green: 18–30, orange: 31–50, blue: 50+).

The mean absolute error (MAE) values for each age group are presented in Table 5a–c and Table 6a–c and Figure 4a–c. Systolic BP was better predicted by telomere measurements only in group 1 (*p* = 0.043). Neither of the used parameters had a better prediction power for SBP in groups 2 and 3 (*p* > 0.05). In addition, there was no difference in prediction power of the used variables (single or in combination) for DBP in either of the study groups (*p* > 0.05, Table 6a–c).

## 4. Discussion

Our results show, for the first time, that the assessment of retinal vascular function, singularly or in combination with RTL, represents a better predictor of chronological age than RTL in individuals under 30 years of age, but not in those older. Indeed, and surprisingly, although there was a trend pointing to its possible predictive power in individuals over 50-years-old, RTL alone was not a good predictor of chronological age in either of our study groups.

In addition, we were also able to demonstrate that, despite correlating negatively with SBP in individuals over 30 years of age, RTL was a better predictor for SBP individuals in subjects less than 30 years old only, while retinal vascular function was not a good predictor for BP in either of the study groups.

In line with our previous reports, in the present study we have yet again demonstrated that, in healthy individuals, retinal vascular function parameters are affected by chronological age [45]. In addition, and in agreement with previous publications [52,53,54], we have also shown that RTL values are declining with age. Nevertheless, our RTL measurements alone had no predictive power for chronological age in either of the study groups. This could have few explanations. It has been suggested that measures of telomere length can be affected by various noise and are not a useful measure of biological age [11]. Indeed, the process of RTL shortening with age is very complex and the exact mechanisms underlying this process are not yet established [55,56,57]. Therefore, it has been proposed that, as the hallmarks of ageing are “multifaceted”, combinations of predictors should be used instead of single ones. Our study also revealed that, in younger individuals (group 1), even when it comes to predicting the actual chronological age, the largest error was given when using the RTL measurements only. A much better prediction was obtained, however, when the RTL values were combined with the retinal vascular function. Nevertheless, what was particularly noticeable was that the prediction error was the lowest when using retinal arterial function measures alone and only in younger individuals. No such relationships were found in either of the other study groups. This is an interesting observation. Presently, significant efforts are put into developing robust predictors of biological ageing, predictors that can be measured quickly and non-invasively. In the light of our findings, retinal vascular function could be such a candidate. Nevertheless, its predictive power for chronological age seems to be limited to only individuals younger than 30 years of age. There is no clear explanation for this finding and more research needs to be conducted in order to understand the exact mechanism behind this observation. We can hypothesise, however, that in older individuals, the influence of genetic, as well as of the long-standing various environmental factors, will result in a larger variability when it comes to assessing their vascular health or attempting to use such parameters as predictors of their age.

The level of BP is another risk factor for CVD, and many attempts were made to possibly predict it, even using retinal vascular photographs [58]. Nevertheless, these methods have serious limitations, such as the fact that in static imaging, there are already evident macroscopic structural changes that show an already present vascular pathology. Therefore, studying function, rather than structure, offers the advantages of detecting early, subclinical vascular pathologies. However, and surprisingly, retinal vascular function was not a good predictor for the measured systemic BP parameters in either of our study groups presented here. Indeed, in the present sample, and also only in the youngest individuals, RTL had the best predictive power for the level of BP. This is a very interesting discovery considering that the close relationship between early stage of hypertension and microvascular retinal dysfunction has already been demonstrated [20,21,46,59]. Moreover, although the telomere length has been previously associated with development of hypertension [13], this parameter has never been used to predict BP values in asymptomatic individuals. Therefore, our finding is novel. More research is necessary, however, in order to precisely understand this.

In summary, it is important to emphasize that, although there is a very high need of predicting CVD risk in asymptomatic individuals, most of the current efforts are still directed towards diagnosing and managing diseases that are already symptomatic. This trend is, probably, the result of the current difficulty in finding good risk predictors that are non-invasive and can also be used in primary care. In young, healthy individuals, this task seems to be easier. Indeed, in this category, the assessment of biological ageing could serve as a good marker for CVD prediction. However, the challenge still remains in the case of older subjects, where many heterogenous and individual-specific vascular physiological and anatomical changes have already occurred. In addition, the challenge is even greater in those where concurrent multi-morbidities or medications affect any attempts at prediction. As a result, the quest for precise markers that can be used to assess biological ageing in individuals of all age groups and CVD risk stratification, is still on.

## Figures and Tables

**Table 1:** General characteristics of the study population.

Variables	Age Group (1) (19–30 Year)	Age Group (2) (31–50 Year)	Age Group (3) (>50 Year)	*p*-Value	Post Hoc Analysis
Number	40	47	36	>0.05	-
Gender	20M:20F	23M:24F	19M:17F	>0.05	-
Age (years)	24.95 (0.72)	38.28 (0.67)	58.53 (0.76)	0.0000 *	1 < 2 < 3
SBP	114.9 (1.84)	114.96 (1.70)	121.86 (1.94)	0.0129 *	1 = 2 < 3
DBP	66 (1.35)	70.64 (1.25)	72.83 (1.43)	0.0022 *	1 = 2 < 3
MAP	82.3 (1.87)	84.6 (1.92)	89.1 (1.98)	0.004 *	1 = 2 < 3
HR (bpm)	66.05 (1.32)	64.94 (1.22)	62.11 (1.39)	0.1133	-
BMI (kg/m^2^)	24.92 (0.71)	26.07 (0.65)	26.53 (0.76)	0.2727	-
Glucose	4.43 (0.11)	4.68 (0.12)	5.00 (0.12)	0.0034 *	1 = 2 < 3
TG (mmol/L)	0.83 (0.050)	0.92 (0.047)	0.98 (0.05)	0.1269	-
T-CHOL	3.94 (0.12)	4.63 (0.12)	4.51 (0.14)	<0.001 *	2 = 3 > 1
HDL-C (mmol/L)	1.33 (0.06)	1.26 (0.06)	1.16 (0.06)	0.051	-
LDL-C (mmol/L)	2.21 (0.13)	3.13 (0.12)	2.92 (0.14)	0.001 *	2 = 3 > 1
GSH	348.79 (47.91)	412.13 (41.03)	410.09 (46.52)	0.549	-
GSSG	31.19 (3.43)	36.80 (2.93)	28.70 (3.33)	0.169	-
RTL	0.64 (0.22)	0.09 (0.21)	−0.36 (0.24)	0.010 *	1 > 2 > 3

SBP, systolic blood pressure; DBP, diastolic blood pressure; HR, heart rate (in beats per minute); BMI, body mass index; GLUC, glucose; TG, triglycerides; T-CHOL, total cholesterol; HDL-C, high-density lipoprotein cholesterol; LDL-C, low-density lipoprotein cholesterol; GSH, glutathione; GSSG, glutathione disulphide; RTL, leucocyte telomere relative length. * Significant *p*-values are indicated where *p* < 0.05 was considered significant. Data are presented as mean (SD) unless otherwise indicated.

**Table 2:** Summary of retinal arterial vascular function parameters.

	Mean (SD)				
Parameter	Age Group (1) (19–30 Years)	Age Group (2) (31–50 Years)	Age Group (3) (>50 Years)	*p*-Value	Post Hoc Analysis
Artery baseline	125.71 (5.02)	114.86 (2.22)	106.46 (6.07)	0.107	
Artery-BDF	6.34 (0.41)	5.22 (0.38)	4.80 (0.44)	0.029	
Artery-DA ^a^	10.80 (0.70)	9.94 (0.66)	8.18 (0.76)	0.041	
Artery-BCFR ^b^	4.48 (0.40)	4.69 (0.37)	3.26 (0.43)	0.034	
Artery-MD	124.11 (2.04)	118.36 (1.90)	116.11 (2.15)	0.021	
Artery-tMD	17.44 (0.58)	17.55 (0.53)	19.89 (0.61)	0.005 *	1 = 2 < 3
Artery-MD%	5.31 (0.32)	4.66 (0.29)	4.30 (0.34)	0.104	-
Artery-MC	113.17 (2.30)	109.92 (2.12)	112.80 (2.43)	0.521	-
Artery-tMC	24.65 (2.49)	23.94 (1.13)	30.75 (3.11)	0.007 *	1 = 2 < 3
Artery-MC%	−3.40 (0.30)	−3.55 (0.27)	−2.70 (0.30)	0.010 *	1 = 2 > 3
Artery-Slope_AD_^c^	0.45 (0.04)	0.39 (0.04)	0.38 (0.05)	0.567	-
Artery-Slope_AC_^c^	−0.56 (0.04)	−0.44 (0.04)	−0.36 (0.05)	0.126	-

BDF, baseline diameter fluctuation; DA, dilation amplitude; BCFR, baseline-corrected flicker response; MD, artery maximum dilation; tMD, reaction time to maximum dilation diameter; MD%, percentage change in diameter from baseline to maximum dilation; MC, artery maximum constriction; tMC, reaction time to maximum constriction diameter from maximum dilation diameter; MC%, percentage constriction below baseline; Slope_AD_, slope of arterial dilation; Slope_AC_, slope of arterial constriction. Unless otherwise indicated, all values are expressed in arbitrary units, which approximately correspond to micrometres (μm) in a normal Gullstrand eye. * Significant *p*-values are indicated where *p* < 0.05 was considered significant. ^a^ Calculated as MD − MC, ^b^ Calculated as DA − BDF, ^c^ Calculated as (MD − baseline)/tMD, ^d^ Calculated as (MC − MD)/tMC.

**Table 3:** Artery measurements and telomere encodings for the symbolic regression formulas.

Feature Representation	Corresponding Measurements
X0	RTL
X1	Artery baseline
X2	Artery Baseline Diameter Fluctuation
X3	Artery Maximum Dilation
X4	Artery Time to Maximum Dilation
X5	Artery Maximum Dilation Percentage
X6	Artery Maximum Constriction
X7	Artery Time to Maximum Constriction
X8	Artery Maximum Constriction Percentage
X9	Artery Dilation Amplitude
X10	Artery Baseline Corrected Flicker Response
X11	Artery Dilation Slope
X12	Artery Constriction Slope

**Table 4:** (**a**) Mean absolute errors for age predictions (Age group 1). (**b**) Mean absolute errors for age predictions (Age group 2). (**c**) Mean absolute errors for age predictions (Age group 3).

Arteries + Telomere		Arteries Only		Telomere Only	
**(a)**					
**Fold**	**MAE**	**Fold**	**MAE**	**Fold**	**MAE**
1	1.488355	1	1.457137	1	2.150139
2	2.03976	2	1.628655	2	2.466321
3	1.634165	3	1.580925	3	3.263452
4	2.157625	4	2.038026	4	3.874383
5	1.157639	5	1.40857	5	1.776776
Average	1.695509	Average	1.622663	Average	2.706214
**(b)**					
**Fold**	**MAE**	**Fold**	**MAE**	**Fold**	**MAE**
1	2.812663	1	2.527317	1	3.204149
2	2.107845	2	2.245985	2	1.518323
3	2.790105	3	2.620622	3	3.189129
4	3.892265	4	3.516084	4	4.30067
5	2.632783	5	2.965419	5	3.701804
Average	2.847132	Average	2.775085	Average	3.182815
**(c)**					
**Fold**	**MAE**	**Fold**	**MAE**	**Fold**	**MAE**
1	4.902158	1	6.013113	1	2.392696
2	2.922922	2	5.230758	2	4.52436
3	4.003612	3	3.691483	3	3.489574
4	4.052891	4	5.85124	4	4.656914
5	6.184841	5	4.847482	5	3.400724
Average	4.413285	Average	5.126815	Average	3.692854

**Table 5:** (**a**) Mean absolute errors for systolic BP predictions (Age group 1). (**b**) Mean absolute errors for systolic BP predictions (Age group 2). (**c**) Mean absolute errors for systolic BP predictions (Age group 3).

Arteries and Telomere		Arteries Only		Telomere Only	
**(a)**					
**Fold**	**MAE**	**Fold**	**MAE**	**Fold**	**MAE**
1	7.60824	1	9.781193	1	9.214715
2	14.67967	2	15.0163	2	8.569096
3	8.132338	3	6.466947	3	9.16523
4	8.570601	4	14.36258	4	6.129705
5	13.69032	5	7.829224	5	9.043941
Average	10.53623	Average	10.69125	Average	8.424537
**(b)**					
**Fold**	**MAE**	**Fold**	**MAE**	**Fold**	**MAE**
1	8.581326	1	8.418168	1	7.495257
2	7.755753	2	7.490878	2	7.130104
3	10.37444	3	7.814303	3	5.463688
4	10.96121	4	8.422463	4	10.27759
5	8.381083	5	6.844803	5	8.355181
Average	9.210761	Average	7.798123	Average	7.744364
**(c)**					
**Fold**	**MAE**	**Fold**	**MAE**	**Fold**	**MAE**
1	17.1292	1	16.13368	1	12.3724
2	6.913672	2	5.783934	2	5.320442
3	8.221233	3	8.26078	3	8.073649
4	8.246475	4	6.949804	4	5.237078
5	7.398514	5	5.531346	5	6.151203
Average	9.581819	Average	8.531909	Average	7.430954

**Table 6:** (**a**) Mean absolute errors for diastolic BP predictions (Age group 1). (**b**) Mean absolute errors for diastolic BP predictions (Age group 2). (**c**) Mean absolute errors for diastolic BP predictions (age group 3).

Arteries and Telomere		Arteries Only		Telomere Only	
**(a)**					
**Fold**	**MAE**	**Fold**	**MAE**	**Fold**	**MAE**
1	5.110043	1	6.43493	1	6.644792
2	6.032743	2	7.535935	2	5.476441
3	4.814686	3	4.070671	3	5.058709
4	4.77134	4	4.221734	4	5.449827
5	4.172079	5	3.580786	5	4.426252
Average	4.980178	Average	5.168811	Average	5.411204
**(b)**					
**Fold**	**MAE**	**Fold**	**MAE**	**Fold**	**MAE**
1	4.733007	1	5.99453	1	4.215411
2	4.618492	2	6.014625	2	5.779917
3	7.849373	3	8.224371	3	5.561008
4	6.65106	4	8.225318	4	5.896184
5	2.782652	5	3.812176	5	8.340123
Average	5.326917	Average	6.454204	Average	5.958529
**(c)**					
**Fold**	**MAE**	**Fold**	**MAE**	**Fold**	**MAE**
1	8.175021	1	7.292121	1	7.451285
2	6.597758	2	5.685044	2	6.589668
3	4.907752	3	5.379021	3	7.05904
4	3.739657	4	5.28883	4	4.040507
5	5.058974	5	6.01973	5	3.199624
Average	5.695832	Average	5.932949	Average	5.668025

## Data Availability

Not applicable.
